# Comparison of PCR-based detection of chromogranin A mRNA with traditional histological lymph node staging of small intestinal neuroendocrine neoplasia

**DOI:** 10.1186/1756-0500-5-318

**Published:** 2012-06-21

**Authors:** Ben Lawrence, Barton Kenney, Bernhard Svejda, Simon Schimmack, Daniele Alaimo, Andrea Barbieri, Jaroslaw Jedrych, Mark Kidd, Irvin Modlin

**Affiliations:** 1 Gastrointestinal Pathobiology Research Group, Yale University School of Medicine, 208602, New Haven, CT, USA; 2 Department of Pathology, Division of Gastrointestinal and Hepatic Pathology, Yale University School of Medicine, 208023, New Haven, CT, USA

**Keywords:** Chromogranin A, Immunohistochemistry, Histopathology, Lymph node, Metastasis, Micrometastasis, Neuroendocrine tumor, Neuroendocrine neoplasm, RNA, Staging

## Abstract

**Background:**

Accurate neuroendocrine neoplasia (NEN) staging is vital for determining prognosis and therapeutic strategy. The great majority of NENs express chromogranin A (CgA) which can be detected at a protein or transcript level. The current standards for lymph node metastasis detection are histological examination after Hematoxylin and Eosin (H&E) and CgA immunohistochemical (IHC) staining. We hypothesized that detection of CgA mRNA transcripts would be a more sensitive method of detecting these metastases.

**Findings:**

We compared these traditional methods with PCR for CgA mRNA extracted from formalin fixed paraffin embedded slides of lymph nodes (*n* = 196) from small intestinal NENs, other gastrointestinal cancers and benign gastrointestinal disease. CgA PCR detected significantly more NEN lymph nodes (75%) than H&E (53%) or CgA IHC (57%) (*p* = 0.02). PCR detected CgA mRNA in 50% (14 of the 28) of SI-NEN lymph nodes previously considered negative. The false positive rate for detection of CgA mRNA was 19% in non-neuroendocrine cancers, and appeared to be due to occult neuroendocrine differentiation or contamination by normal epithelium during histological processing.

**Conclusions:**

Molecular pathological analysis demonstrates the limitations of observer-dependent histopathology. CgA PCR analysis detected the presence of CgA transcripts in lymph nodes without histological evidence of tumor metastasis. Molecular node positivity (stage _mol_N1) of SI-NEN lymph nodes could confer greater staging accuracy and facilitate early and accurate therapeutic intervention. This technique warrants investigation using clinically annotated tumor samples with follow-up data.

## Findings

### Research hypothesis

The prognosis of solid malignancies is inextricably linked to the extent of invasion and spread from the site of the primary tumor, known as the “tumor stage”. Tumors that exhibit metastases to regional lymph nodes have a worse prognosis than tumors that remain localized within the primary organ, since cure is more likely after surgery for localized disease. Tumor lymph node metastases are usually identified during examination of resected lymph nodes on paraffin embedded histological slides stained with Hematoxylin and Eosin (H&E), and the detection of tumor cells in ambiguous cases is aided by immunohistochemical (IHC) staining for the tumor marker protein Chromogranin A (CgA).

An alternative objective technique for assessment of lymph node metastasis is Quantitative Real Time PCR. This technique has been previously used to detect histologically invisible ‘micrometastases’ by identifying the expression of tumor marker RNA transcripts in lymph nodes resected in the setting of non-neuroendocrine cancers [1-9]. We hypothesized that a PCR-based approach for CgA detection would be more sensitive than either H&E or CgA IHC for detecting lymph node metastases.

### Methods

Study tissue was stored and collated according to the requirements of the Institutional Review Board at Yale New Haven Hospital. All resections for SI-NENs between the years 2000 and 2010 were included in the study. Control tissue was obtained from lymph nodes extracted at the time of resection of non-neuroendocrine malignancy or non-malignant conditions in organs which exhibit similar mesenteric lymph node drainage as SI-NENs. Three contiguous histological slides were randomly assigned to H&E staining, CgA IHC, or RNA extraction for CgA PCR (PCR).

H&E and IHC staining of the paraffin-embedded slides were conducted using standard clinical pathological methodology. IHC staining used the Chromogranin A primary antibody, MS-382 (NeoMarkers, Thermo Scientific, Fremont, CA) applied at a 1:1000 concentration. Slides assigned to CgA PCR were processed by scraping tumor tissue from unstained paraffin embedded slides and RNA extracted using QuickExtract^TM^ FFPE RNA Extraction Kit (Epicentre Biotechnologies, Madison, WI). Total RNA from each sample was subjected to reverse transcription with the High Capacity cDNA Archive Kit (ABI, Foster City, CA). The method included 2 μg of total RNA in 50μL of water mixed with 50μL of 2X RT mix containing Reverse Transcription Buffer, dNTPs, random primers and Multiscribe Reverse Transcriptase. RT reaction was carried out in a thermal cycler for 10 minutes at 25 °C followed by 120 minutes at 37 °C. Quantitative Real-time PCR (qRT-PCR) analysis was then performed in duplicate [10] using the ABI 7900 Sequence Detection System (Applied Biosystems, Foster City, CA). We mixed cDNA in 7.2μL of water with 0.8μL of 20X Assays-on-Demand primer and probe mix and 8μL of 2X TaqMan Universal Master mix in a 384-well optical reaction plate. The following PCR conditions were used: 50 °C for 2 minutes, then 95 °C for 10 minutes, followed by 40 cycles at 95 °C/15 seconds and 60 °C/60 seconds. A standard curve was generated for each gene using cDNA obtained by pooling equal amounts from each sample. Detection of CgA by qRT-PCR was conducted using 3 separate and non-overlapping primers (Applied Biosystems, Foster, CA) to achieve representative coverage of the 8 exon CgA gene (CHGA Hs00900369_s1, Hs00900371_s1, Hs00900373). The expression level of target genes was normalized to internal 18S; the presence of other housekeeping genes ALG9 and GAPDH was examined but was not utilized in calculations because these two genes were not consistently detectable in the tumor samples. The coefficient of variation for cycle time was similar between 18S and the three Chromogranin A target genes (18S 10.37%, CgA_69 14.46%, CgA_71 13.07%, CgA_73 13.93%). Detection of CgA by qRT-PCR was conducted using 3 separate and non-overlapping primers (Applied Biosystems, Foster, CA) to achieve representative coverage of the 8 exon CgA gene (CHGA Hs00900369_s1, Hs00900371_s1, Hs00900373). Lymph nodes were considered positive for the presence of CgA by PCR if two criteria were met: one (or more) of the three primers detected expression of that primer, and expression was 5-fold increased relative to lymph nodes from benign gastrointestinal disease. Three pathologists independently examined the 388 stained slides presented in a random order and determined whether the lymph node was positive for NEN metastasis. Following this rating step, one pathologist (BK) examined each slide to quantify the extent of tumor involvement and the presence of non-malignant non-lymphoid normal tissue.

A power analysis (α 0.05, power of 0.80, Glass’s delta 0.56, *sd* 0.49) was conducted prior to sample collection based on a pilot study of 22 SI-NEN lymph nodes which determined a sample size of 52 per group.

### Results

**Figure 1:**
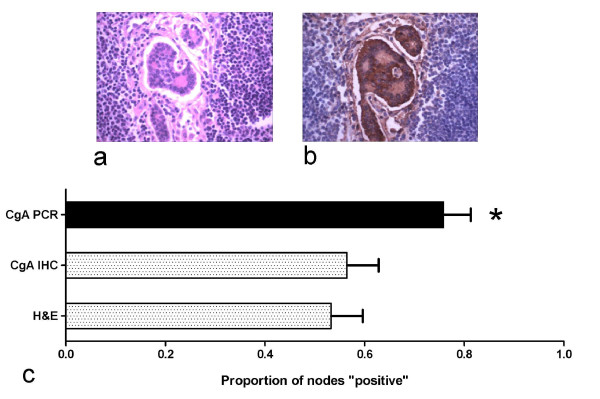
**Examples of NEN metastasis and the proportion of regional lymph nodes that exhibit histological tumor involvement, Chromogranin A immunohistochemical positivity or Chromogranin A transcript expression.** A NEN lymph node metastasis stained with H&E stain, 20X (1**a**) and Chromogranin A IHC stain, 20X (1**b**) on contiguous slices of an involved lymph node. There was a significant difference between the proportion of positive tumors (*p* = 0.020), and Dunn’s post hoc tests showed a significant difference between H&E staining or CgA PCR (1**c**).

The sample included 61 lymph nodes from 21 patients with NENs (*n* = 20 SI-NENs, *n* = 1 gastric NEN), 115 lymph nodes from 19 patients with non-neuroendocrine cancers, and 19 lymph nodes from 11 patients with non-malignant disease. Concordance between the three pathologists regarding neuroendocrine tumor involvement was 96.4% on H&E staining and 97.4% on CgA IHC. In NEN regional lymph nodes, CgA transcripts (77%, 47/61) were detected significantly more frequently than tumor cells on H&E staining (Figure 1a; 54%, 33/61) or CgA protein (Figure 1b; 56%, 34/61) on IHC staining (see Figure 1c; *p* = 0.02, post-hoc testing H&E vs PCR significant). Two lymph nodes that were positive on H&E staining were negative for CgA transcript, making a total of 49/61 (80.3%) positive nodes on H&E and PCR combined, compared to 33/61 (54.1%) on H&E alone (*p* = 0.003). Considering the 21 NEN cases on a patient-by-patient basis, 9 had at least one H&E negative node upstaged to positive by PCR. Of the three patients with localized disease (TxN0M0), two were upstaged to lymph node positive disease (TxN1M0) by PCR analysis (Table 1), and one patient with distant metastases was upstaged from node-negative to node-positive. Of the 21 NEN cases, 19 patients remain alive, thus preventing a prognostic analysis of survival.

**Table 1:** Staging of SI-NENs according to histological and PCR-based analyses

	**H&E Node positive**	**PCR Node positive**	**Percent upstaged by PCR**
No Distant Metastases	6 / 9	8 / 9	22%*
Distant Metastases	11 / 12	12 / 12	8%
Total	17 / 21	20 / 21	14%

*Upstaging by TNM criteria.

The 21 SI-NENs included 3 patients with localized disease, 6 with nodal metastasis according to H&E assessment, and 12 with distant metastases (one was lymph node negative). PCR upstaged 2 of the 3 node negative patients to node positive, and the patient with node negative but metastatic disease was also node positive on PCR. Thus 3 of 21 (14%) patients were upstaged to node positive disease, with 2 patients upstaged in TNM staging.

**Figure 2:**
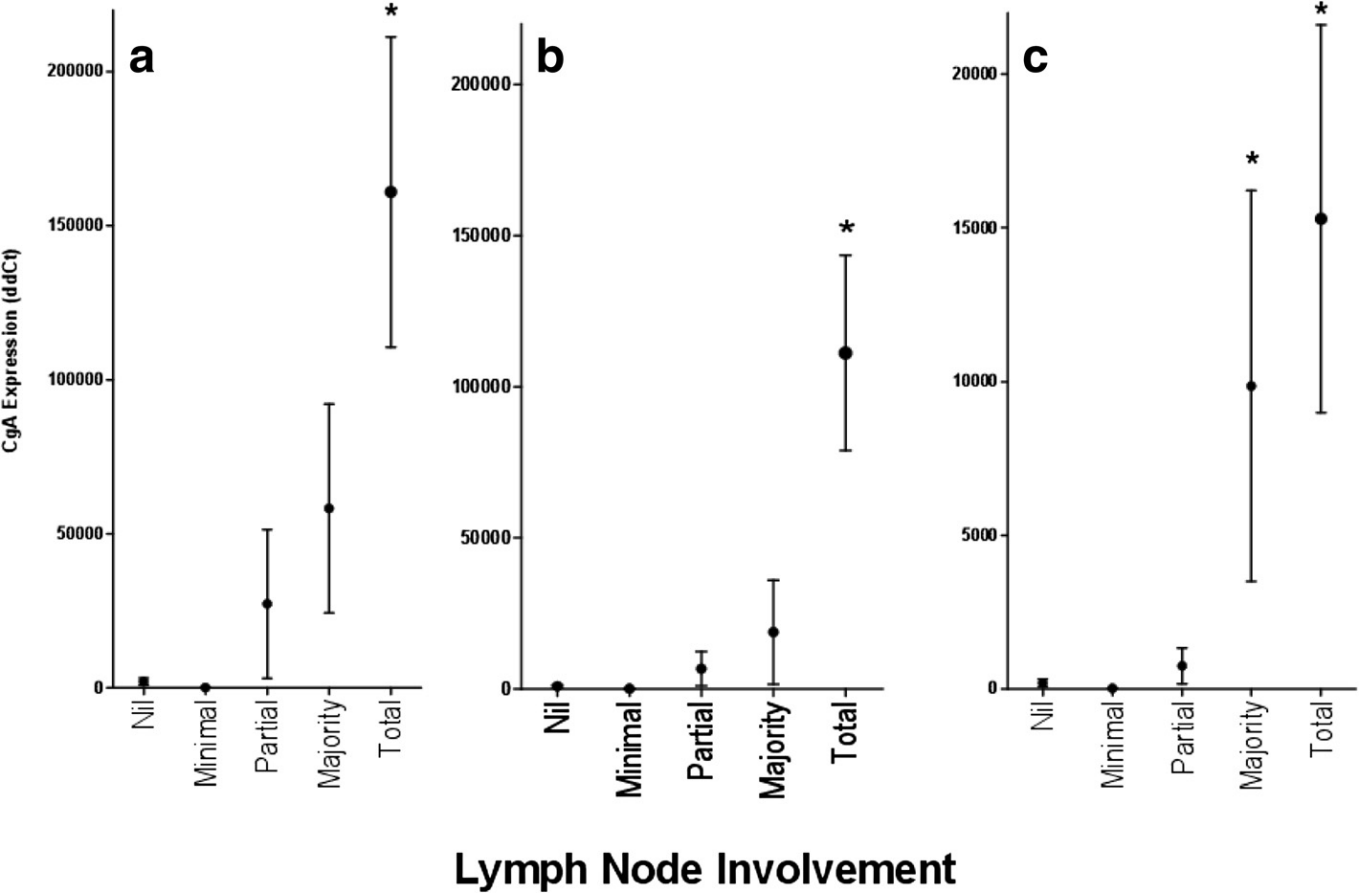
**Chromogranin A transcript expression level of and degree of lymph node involvement by tumor.** The mean (and SEM) expression level of 3 different CgA primers, namely CHGA Hs00900369_s1 (2**a**), CHGA Hs00900371_s1 (2**b**), and CHGA Hs00900373_s1 (2**c**) is shown. The expression level of CgA differs significantly in all three primers (*p* < .0001, *p* = .0004 and *p* < .0001 respectively). Post-hoc testing shows significantly higher expression in the ‘total replacement’ group than in the ‘nil’ or ‘minimal tumor’ groups for all primers. In addition, post hoc tests show a significant difference between ‘Nil’ and ‘partial replacement’ in the primer shown in 2c.

Validation of a quantitative relationship between CgA transcripts and lymph node involvement was investigated. The level of transcript expression was correlated with the degree of tumor involvement of lymph nodes across all three CgA primers (Figure 2), with higher levels of CgA expression seen in lymph nodes with majority or complete replacement by NEN.

**Figure 3:**
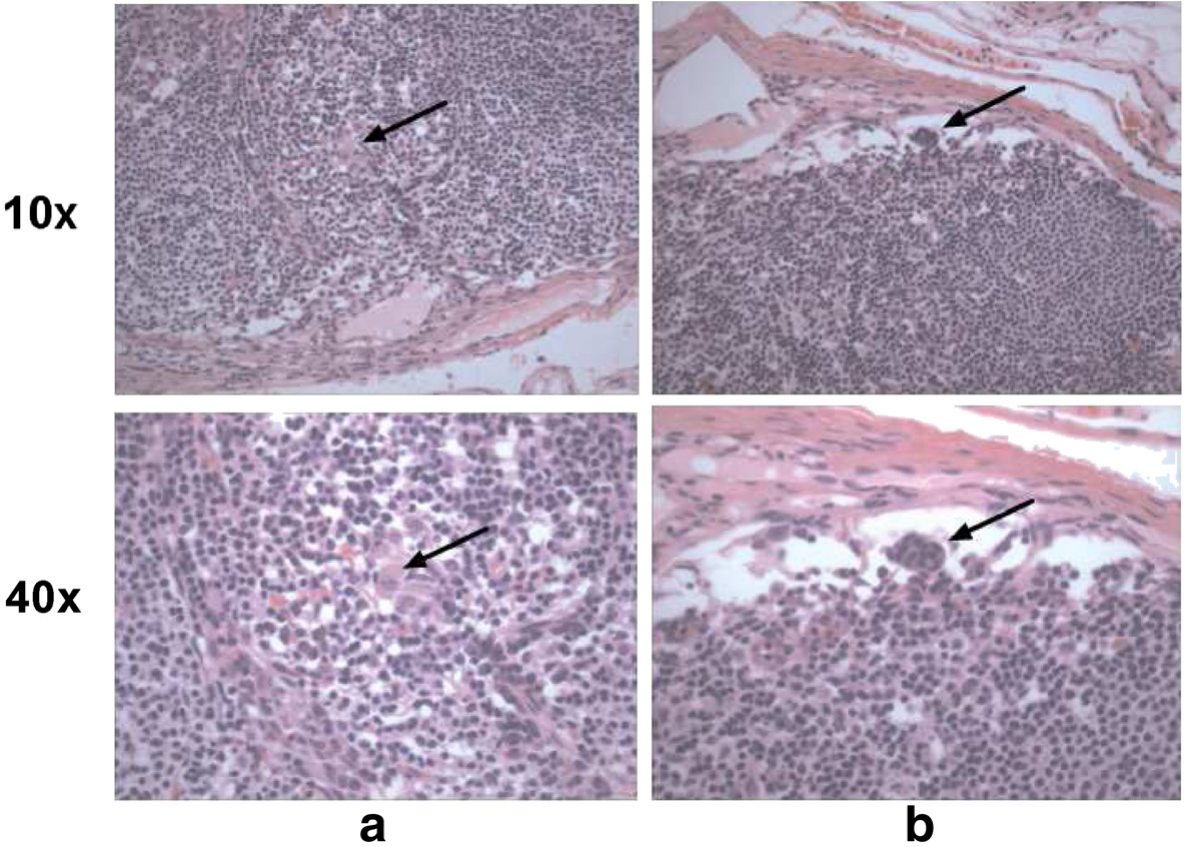
**Suspicious “tumor” cell clusters in lymph nodes that are negative for tumor metastasis on examination after H&E staining but positive for CgA RNA after PCR of lymph node tissue.** The arrows point to suspicious minute cell clusters in case (3**a**) and (3**b**), shown under lower and higher magnification.

Of the 28 lymph nodes without histological tumor involvement, 14 had detectable CgA by PCR. These cases were carefully retrospectively reviewed (following un-blinding) for possible NEN involvement. Many cases had rare/subtle suspicious cells seen on high magnification which were likely to be below the threshold for H&E diagnosis of metastasis. (Examples are shown in Figure 3).

**Figure 4:**
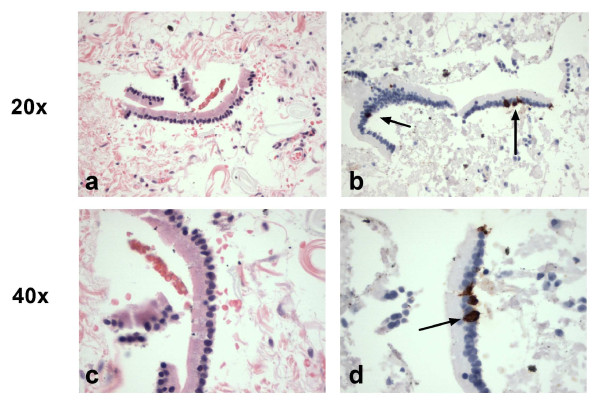
**Examples of epithelial contamination of lymph nodes extracted with non-neuroendocrine malignancies, presumably caused during sample processing.** These figures show small fragments of epithelium after H&E staining (4**a** and 4**c**) and matching epithelium in contiguous slides after Immunostaining for CgA (4**b** and 4**d**) in lymph node samples that exhibit PCR positivity. These tiny pieces of normal epithelium contain normal neuroendocrine cells (indicated by arrows) and therefore CgA transcripts are detectable. The top row is shown at 20x magnification and the lower row at 40x magnification.

The rate of CgA mRNA positivity in the lymph nodes resected with non-neuroendocrine cancers was 29 of 115 (25%). Of the 29 lymph nodes with detectable CgA transcript from the non-neuroendocrine malignancies, 7 nodes belonged to three patients with adenocarcinomas (pancreatic, rectal, small intestinal) that had positive CgA IHC or histological evidence of neuroendocrine differentiation, and so should be regarded as exhibiting neuroendocrine differentiation and the CgA PCR result are interpreted as a true positive in terms of tumor phenotype. The non-neuroendocrine cancers were then examined on a case-by-case basis to determine whether the distribution of the remaining 22 (19.1%) lymph nodes with detectable CgA transcripts was related to characteristics of the resected tumor (Additional file 1: Table S1A). There was no relationship between clinical variables and the presence of CgA mRNA; transcript detection did not relate to age, gender, stage, grade, or primary site. Rather, the low level of lymph node positivity detected across the majority of non-neuroendocrine cancers raised the possibility that a more general and non-prognostic phenomenon might account for this finding. Therefore, the presence and nature of extraneous tissue other than lymph node or metastasis was quantified for all 195 lymph nodes by examination of the H&E slide. Nearly one half of the slides contained additional tissue adjacent to (or sometimes within) the lymph node (Additional file 1: Table S1B), but there was no relationship between the type of contamination on the slide and the likelihood of CgA transcript detection. The most common contaminant was microscopic fragments of epithelium from small bowel. These strips of epithelium contained cells with IHC positive staining for CgA, suggesting that CgA transcript detection could occur in absence of NEN metastasis by the detection of normal neuroendocrine cells exogenously introduced by mucosal or epithelial contamination during histological processing (Figure 4). This type of systematic error in processing was also suggested by the similar frequency of transcript positivity between non-neuroendocrine cancer and benign diseases (19% and 21% respectively). There were also some cases of macroscopic pancreatic or bowel wall tissue on the slide.

### Conclusions

In this study, regional mesenteric lymph nodes from resected SI-NENs were examined for the histological presence of metastasized NEN cells, the immunohistochemical presence of CgA protein, or the presence of CgA mRNA by PCR. CgA transcripts were detected at a significantly higher rate than NEN cells by examination after H&E or CgA IHC staining. Since it is evident that anti neoplastic therapy is more effective as tumor burden decreases [11], and the presence of lymph node metastases has been validated as a key prognostic variable in NENs [12,13] the identification of lymph node metastasis has significant implications on the timing of therapy and its efficacy. An issue awaiting clarification is the significance of CgA transcripts detected in lymph nodes from some non-neuroendocrine tumors, which could represent either occult neuroendocrine differentiation (24% exhibited histological or IHC evidence of neuroendocrine differentiation) or false positive tumor detection. The detection of CgA mRNA could represent contamination by normal neuroendocrine cells contained in the gastrointestinal mucosa, although there was no difference in CgA transcript detection between slides contaminated with epithelial versus non-epithelial cells to support this. On the other hand, there has been considerable discussion about NE cells as the origin of some GI cancers and this may represent the detection of alterations in cell lineage in evolution of an adenocarcinoma [14-16].

We have demonstrated that the PCR detection of CgA transcripts from paraffin embedded lymph node tissue is a feasible technique with potential clinical utility. Validation in a large sample of clinically annotated tumors is therefore warranted.

### Availability of supporting data

Additional file 1 Table S1A and S1B are included are available at [hyperlink].

## Abbreviations

CgA, Chromogranin A; H&E, Hematoxylin and eosin; IHC, Immunohistochemistry; NEN, Neuroendocrine neoplasm (formerly known as NET [Neuroendocrine Tumor]); PCR, Polymerase chain reaction; SI-NEN, Small intestinal neuroendocrine neoplasm.

## Competing interests

The authors declare that they have no competing interests.

## Authors’ contributions

BL; study design, qRT-PCR, statistical analysis, manuscript preparation. BK; study conception, study design, pathological examination of tissue. BS; study design, histopathological and immunohistochemical assessment, manuscript preparation. SS; qRT-PCR and design of analysis. DA; qRT-PCR, manuscript preparation. AB; histopathological and immunohistochemical assessment. JJ; histopathological and immunohistochemical assessment. MK; study conception, analysis and design. IM; study design, manuscript preparation. All authors read and approved the final manuscript.

## Supplementary Material

Additional file 1 **Table S1A.** The traditional prognostic characteristics of the non-neuroendocrine cancers and the proportion of lymph nodes with Chromogranin A transcript detection. The distribution of PCR positive lymph nodes occurred irrespective of primary site, differentiation, grade and stage. **Table S1B.** Proportion of lymph nodes that show detectable CgA transcripts in the absence of histological evidence of neuroendocrine tumor according to the presence of extraneous tissue. There was no relationship between the nature of contamination and the likelihood of CgA transcript detection, and in particular, the presence of epithelial cell “contamination” did not increase the detection of CgA transcripts. Also, 3 of the 6 lymph nodes with adenocarcinoma cells visible on histological examination also demonstrated CgA IHC positivity, consistent with detection of neuroendocrine differentiation by CgA PCR. Click here for file

## References

[B1] Qiu Y Yang H Chen H Ge L Xu X Xiong X He J Detection of CEA mRNA, p53 and AE1/AE3 in haematoxylin-eosin-negative lymph nodes of early-stage non-small cell lung cancer may improve veracity of N staging and indicate prognosis Jpn J Clin Oncol 2010 40 146 152 10.1093/jjco/hyp144 19897851

[B2] Sonoda H Yamamoto K Kushima R Okabe H Tani T Detection of lymph node micrometastasis in gastric cancer by MUC2 RT-PCR: usefulness in pT1 cases J Surg Oncol 2004 88 63 70 10.1002/jso.20143 15499573

[B3] Soikkeli J Lukk M Nummela P Virolainen S Jahkola T Katainen R Harju L Ukkonen E Saksela O Holtta E Systematic search for the best gene expression markers for melanoma micrometastasis detection J Pathol 2007 213 180 189 10.1002/path.2229 17891747

[B4] Shores CG Yin X Funkhouser W Yarbrough W Clinical evaluation of a new molecular method for detection of micrometastases in head and neck squamous cell carcinoma Arch Otolaryngol Head Neck Surg 2004 130 937 942 10.1001/archotol.130.8.937 15313863

[B5] Ho SB Hyslop A Albrecht R Jacobson A Spencer M Rothenberger DA Niehans GA D'Cunha J Kratzke RA Quantification of colorectal cancer micrometastases in lymph nodes by nested and real-time reverse transcriptase-PCR analysis for carcinoembryonic antigen Clin Cancer Res 2004 10 5777 5784 10.1158/1078-0432.CCR-03-0507 15355906

[B6] Kowalewska M Radziszewski J Kulik J Barathova M Nasierowska-Guttmajer A Bidzinski M Pastorek J Pastorekova S Siedlecki JA Detection of carbonic anhydrase 9-expressing tumor cells in the lymph nodes of vulvar carcinoma patients by RT-PCR Int J Cancer 2005 116 957 962 10.1002/ijc.21106 15856466

[B7] Viale G Dell'Orto P Biasi MO Stufano V De Brito Lima LN Paganelli G Maisonneuve P Vargo JM Green G Cao W Comparative evaluation of an extensive histopathologic examination and a real-time reverse-transcription-polymerase chain reaction assay for mammaglobin and cytokeratin 19 on axillary sentinel lymph nodes of breast carcinoma patients Ann Surg 2008 247 136 142 10.1097/SLA.0b013e318157d22b 18156933

[B8] Kuo CT Hoon DS Takeuchi H Turner R Wang HJ Morton DL Taback B Prediction of disease outcome in melanoma patients by molecular analysis of paraffin-embedded sentinel lymph nodes J Clin Oncol 2003 21 3566 3572 10.1200/JCO.2003.01.063 12913098

[B9] Mocellin S Hoon DS Pilati P Rossi CR Nitti D Sentinel lymph node molecular ultrastaging in patients with melanoma: a systematic review and meta-analysis of prognosis J Clin Oncol 2007 25 1588 1595 10.1200/JCO.2006.09.4573 17443001

[B10] Kidd M Modlin IM Mane SM Camp RL Eick G Latich I The role of genetic markers–NAP1L1, MAGE-D2, and MTA1–in defining small-intestinal carcinoid neoplasia Ann Surg Oncol 2006 13 253 262 10.1245/ASO.2006.12.011 16424981

[B11] Edge S, Byrd D, Compton C, Fritz A, Greene F, Trotti A AJCC Cancer Staging Manual 2010 Springer, New York

[B12] Pape UF Jann H Muller-Nordhorn J Bockelbrink A Berndt U Willich SN Koch M Rocken C Rindi G Wiedenmann B Prognostic relevance of a novel TNM classification system for upper gastroenteropancreatic neuroendocrine tumors Cancer 2008 113 256 265 10.1002/cncr.23549 18506737

[B13] Jann H Roll S Couvelard A Hentic O Pavel M Muller-Nordhorn J Koch M Rocken C Rindi G Ruszniewski P Neuroendocrine tumors of midgut and hindgut origin: Tumor-node-metastasis classification determines clinical outcome Cancer 2011 117 3332 3341 Epub 22011 Jan 258183310.1002/cncr.25855 21246527 10.1002/cncr.25855

[B14] Bakkelund K Fossmark R Nordrum I Waldum H Signet ring cells in gastric carcinomas are derived from neuroendocrine cells J Histochem Cytochem 2006 54 615 621 10.1369/jhc.5A6806.2005 16344325

[B15] Fossmark R Martinsen TC Bakkelund KE Kawase S Waldum HL ECL-cell derived gastric cancer in male cotton rats dosed with the H2-blocker loxtidine Cancer Res 2004 64 3687 3693 10.1158/0008-5472.CAN-03-3647 15150129

[B16] Waldum HL Aase S Kvetnoi I Brenna E Sandvik AK Syversen U Johnsen G Vatten L Polak JM Neuroendocrine differentiation in human gastric carcinoma Cancer 1998 83 435 444 10.1002/(SICI)1097-0142(19980801)83:3<435::AID-CNCR11>3.0.CO;2-X 9690535

