# Supplementary material for: Comparison of PCR-based detection of chromogranin A mRNA with traditional histological lymph node staging of small intestinal neuroendocrine neoplasia
Source: BMC Res Notes. 2012 Jun 21;5:318. doi: 10.1186/1756-0500-5-318 (PMC3441285; doi:10.1186/1756-0500-5-318)
Supplement: Additional file 1 — Table S1A. The traditional prognostic characteristics of the non-neuroendocrine cancers and the proportion of lymph nodes with Chromogranin A transcript detection. The distribution of PCR positive lymph nodes occurred irrespective of primary site, differentiation, grade and stage. Table S1B. Proportion of lymph nodes that show detectable CgA transcripts in the absence of histological evidence of neuroendocrine tumor according to the presence of extraneous tissue. There was no relationship between the nature of contamination and the likelihood of CgA transcript detection, and in particular, the presence of epithelial cell “contamination” did not increase the detection of CgA transcripts. Also, 3 of the 6 lymph nodes with adenocarcinoma cells visible on histological examination also demonstrated CgA IHC positivity, consistent with detection of neuroendocrine differentiation by CgA PCR. [file 1756-0500-5-318-S1.docx]

Supplementary Tables.

**Supplementary Table A**: The traditional prognostic characteristics of the non-neuroendocrine cancers and the proportion of lymph nodes with Chromogranin A transcript detection. The distribution of PCR positive lymph nodes occurred irrespective of primary site, differentiation, grade and stage.

| **Case** | **Primary site** | **Type** | **Differentiation** | **Grade** | **Stage** | **PCR positive** | **Total nodes** |
| --- | --- | --- | --- | --- | --- | --- | --- |
| **1** | Colon | Adenocarcinoma^#^ | moderate/poor | 2 to 3 | T1N1 IIIA | 2 | 8 |
| **2** | Colon | adenocarcinoma | moderate/poor | 2 to 3 | T4N0 IIB | 1 | 8 |
| **3** | Colon | adenocarcinoma | moderate | 2 | T3N0 IIA | 0 | 13 |
| **4** | Colon | adenocarcinoma | moderate | 2 | T3N1 IIIB | 3 | 15 |
| **5** | Colon | adenocarcinoma | moderate | 2 | T4N1M1 IV | 3 | 10 |
| **6** | Pancreas | ductal adenocarcinoma | moderate | 2 | T3N1 IIB | 2* | 3 |
| **7** | Pancreas | anaplastic adenocarcinoma | poor | 4 | T2N0 IB | 1 | 7 |
| **8** | Pancreas | ductal adenocarcinoma | poor | 4 | T3N0 IIA | 3 | 3 |
| **9** | Pancreas | adenosquamous carcinoma | moderate/poor | 3 | T3N0 IIA | 1 | 4 |
| **10** | Pancreas | ductal adenocarcinoma | well/moderate | 2 | T3N1 IIB | 2 | 8 |
| **11** | Pancreas | ductal adenocarcinoma | well/moderate | 2 | T3N0 IIA | 2 | 3 |
| **12** | Rectum | adenocarcinoma | poor | 3 | T3N2 IIIC | 1 | 5 |
| **13** | Rectum | adenocarcinoma | mod | 2 | T3N2 IIIC | 4* | 8 |
| **14** | Small intestine | adenocarcinoma | well/moderate | 1 to 2 | T3N0 II | 2* | 4 |
| **14** | Small intestine | adenocarcinoma | moderate/poor | 2 to 3 | T3N1 III | 0 | 4 |
| **16** | Small intestine | adenocarcinoma | moderate | 2 | T4N1 III | 1 | 5 |
| **17** | Small intestine | adenocarcinoma | moderate | 2 | recurrent | 1 | 4 |
| **18** | Small intestine | Adenocarcinoma (metastasis to colon) | moderate | 2 | T4N0M1b | 0 | 1 |
| **19** | Small intestine | GIST** | N/A | low | N/A | 0 | 1 |
| ***Total*** |  |  |  |  |  | *29* | *115* |

* Included CgA positive nodes assumed to represent neuroendocrine differentiation within the tumor

** GIST = Gastrointestinal Stromal Tumor N/A = Not Applicable

**Supplementary Table B**: Proportion of lymph nodes that show detectable CgA transcripts in the absence of histological evidence of neuroendocrine tumor according to the presence of extraneous tissue. There was no relationship between the nature of contamination and the likelihood of CgA transcript detection, and in particular, the presence of epithelial cell “contamination” did not increase the detection of CgA transcripts. Also, 3 of the 6 lymph nodes with adenocarcinoma cells visible on histological examination also demonstrated CgA IHC positivity, consistent with detection of neuroendocrine differentiation by CgA PCR.

| **Malignancy** | **CgA Transcript** | **Nil** | **Vessels, Fat, Nerves** | **Epithelial Cells** | **Adeno-carcinoma Cells** | **Total H&E negative nodes** |
| --- | --- | --- | --- | --- | --- | --- |
| **NEN** | No | 1 | 4 | 6 | 0 | 11 |
|  | Yes | 7 (88%) | 3 (43%) | 7 (46%) |  | 17 (61%) |
| **Other Cancers** | No | 2 | 47 | 20 | 8 | 77 |
|  | Yes | 0 | 17 (27%) | 12 (38%) | 6 (43%)* | 35 (31%) |

* Three of these 6 lymph nodes exhibited overt CgA IHC positivity.
